# Differences in Nutritional Status and Inflammatory Biomarkers between Female and Male Patients with Bronchiectasis: A Large-Cohort Study

**DOI:** 10.3390/biomedicines9080905

**Published:** 2021-07-28

**Authors:** Xuejie Wang, Carmen Villa, Yadira Dobarganes, Casilda Olveira, Rosa Girón, Marta García-Clemente, Luis Maíz, Oriol Sibila, Rafael Golpe, Rosario Menéndez, Juan Rodríguez-López, Concepción Prados, Miguel Angel Martinez-García, Juan Luis Rodriguez, David de la Rosa, Xavier Duran, Esther Barreiro

**Affiliations:** 1 Lung Cancer and Muscle Research Group, Pulmonology Department, Hospital del Mar-IMIM, Parc de Salut Mar, 08003 Barcelona, Spain; xue62392@gmail.com; 2 Department of Medicine, Universitat Autònoma de Barcelona (UAB), 08035 Barcelona, Spain; 3 Respiratory Department, Clínica Fuensanta, 28015 Madrid, Spain; mcvillac@gmail.com (C.V.); yadira.dobarganes@yahoo.es (Y.D.); 4 Respiratory Department, Hospital Regional Universitario de Málaga, 29003 Málaga, Spain; casi1547@separ.es; 5 Instituto de Investigación Biomédica de Málaga (IBIMA), Universidad de Málaga, 29003 Málaga, Spain; 6 Respiratory Department, Instituto de Investigación Sanitaria, Hospital Universitario de la Princesa, 28015 Madrid, Spain; rmgiron@gmail.com; 7 Respiratory Department, Hospital Universitario Central de Asturias, 33071 Oviedo, Spain; mgclemen@gmail.com; 8 Respiratory Department, Hospital Ramon y Cajal, 28015 Madrid, Spain; luis.maiz@salud.madrid.org; 9 Respiratory Department, Hospital Clínic, 08035 Barcelona, Spain; OSIBILA@clinic.cat; 10 Centro de Investigación en Red de Enfermedades Respiratorias (CIBERES), Instituto de Salud Carlos III (ISCIII), 28015 Madrid, Spain; mianmartinezgarcia@gmail.com; 11 Respiratory Department, Hospital Lucus Augusti, 27080 Lugo, Spain; rafa898@separ.es; 12 Respiratory Department, Hospital Universitario y Politécnico La Fe, 46003 Valencia, Spain; rosmenend@gmail.com; 13 Respiratory Department, Hospital San Agustin, 33405 Avilés, Spain; juan_rodriguezl@hotmail.com; 14 Respiratory Department, Hospital la Paz, 28015 Madrid, Spain; conchaprados@gmail.com; 15 Respiratory Department, Hospital Clínico San Carlos, 28015 Madrid, Spain; jlrhermosa@yahoo.es; 16 Instituto de Investigación Sanitaria del Hospital Clínico San Carlos (IdISSC), 28015 Madrid, Spain; 17 Departament of Medicine, Universidad Complutense de Madrid, 28015 Madrid, Spain; 18 Respiratory Department, Hospital Santa Creu I Sant Pau, 08035 Barcelona, Spain; david.rosa23@gmail.com; 19 Scientific and Technical Department, Hospital del Mar-IMIM, 08035 Barcelona, Spain; xduran@imim.es; 20 Department of Health and Experimental Sciences (CEXS), Universitat Pompeu Fabra (UPF), 08035 Barcelona, Spain

**Keywords:** bronchiectasis, gender-related differences, systemic inflammation, nutritional status, biomarkers, disease severity

## Abstract

We hypothesized that systemic inflammatory and nutritional parameters may differ between male and female patients with non-CF bronchiectasis. In a large patient cohort from the Spanish Online Bronchiectasis Registry (RIBRON), clinical features, systemic inflammatory and nutritional parameters were analyzed in male and female patients with bronchiectasis. Lung function, disease severity using several scores, nutritional status, systemic inflammatory parameters, and multivariate regression analyses were performed to identify differences between male and female patients in the target variables. The number of female patients included in the registry was greater than male patients and they had a less severe disease as measured by all three indices of disease severity, a lower degree of airway obstruction, worse diffusion capacity and airway trapping, better nutritional parameters, and lower levels of inflammatory biomarkers. Multivariate regression analysis evidenced that strong relationships were found between female gender and the following variables: total numbers of leukocytes and neutrophils, hemoglobin, hematocrit, creatinine, and body mass index (BMI). Multivariate regression analyses evidenced that nutritional parameters and inflammatory biomarkers may be reliable indicators of gender-related differences in patients with non-CF bronchiectasis. These findings deserve further attention in follow-up investigations in which the potential predictive value of those biomarkers should be thoroughly explored.

## 1. Introduction

Non-cystic fibrosis (CF) bronchiectasis is a disease characterized by distortion of the airways which entails a range of clinical symptoms among the patients. The etiology of bronchiectasis varies widely from previous tuberculosis and other lung infections to genetic disorders [1,2,3]. Bronchiectasis is also commonly associated with other respiratory diseases, namely asthma and chronic obstructive pulmonary disease (COPD) [4]. Despite the prevalence of bronchiectasis being heterogeneous across geographical regions, recent evidence shows that it is increasing in many countries [4].

Sex-related differences may happen in several respiratory diseases including bronchiectasis [5,6]. Moreover, disease outcomes may also differ between male and female patients with chronic respiratory disorders [7]. Comorbidities, lung anatomy and physiology, chronic infection and inflammation, environmental factors, and altered host defense mechanisms are the most relevant contributors to accounting for the reported gender differences in bronchiectasis patients [6,8]. Anatomical differences in lung and airway structure may also explain the greater susceptibility of female patients to experience lung infections at a younger age [6]. Nutritional abnormalities, delayed diagnosis, and socio-cultural inequality may also favor earlier infections to happen in female patients than in males [9,10]. Whether female patients with bronchiectasis exhibit a greater systemic proinflammatory profile than male patients remain to be fully elucidated.

Phenotypic stratification is key in medical research as it allows for a better characterization of the patients that contribute to tailoring more specific treatments. In this regard, severity indices are useful tools that may help predict mortality [11]. As such, a cut-off value greater than 5 showed to reliably predict hospitalizations and all-cause mortality according to the FACED (FEV_1_, age, chronic colonization, radiological extension, and dyspnea) and bronchiectasis severity index (BSI) scores, as reported in a recent meta-analysis [12]. Furthermore, specific phenotypic characterization of female and male patients is also of relevance in clinical settings for the better management of patients with bronchiectasis. This information is clearly lacking in the literature.

On this basis, we hypothesized that levels of systemic inflammatory parameters may differ between male and female patients with bronchiectasis. Thus, the main objectives were to analyze clinical features including systemic inflammatory cellular and soluble parameters in male and female patients with bronchiectasis in a large-cohort of patients from the Spanish Online Bronchiectasis Registry (RIBRON) [13]. Thus, the specific objectives were to explore potential differences between male and female patients with bronchiectasis as to the following features: (1) lung function, (2) disease severity using several scores, (3) nutritional status, (4) systemic inflammatory parameters, and (5) to assess potential associations between either the inflammatory or the nutritional parameters with the clinical variables in each group of patients. Multiple regression analysis was used to confirm gender differences for the study variables. Moreover, the potential contribution of COPD to the study results was also analyzed in the investigation.

## 2. Materials and Methods

### 2.1. Study Design

This was an observational, cross-sectional, prospective, and multicenter study, in which 43 centers from Spain participated within the frame of the RIBRON database between February 2015 and October 2019 [13]. The quality of the data introduced in the registry was always monitored and ensured by an external contract research organization (CRO). Strengthening the Reporting of Observational Studies in Epidemiology (STROBE) guidelines were followed in this study [14].

### 2.2. Study Population

**Figure 1:**
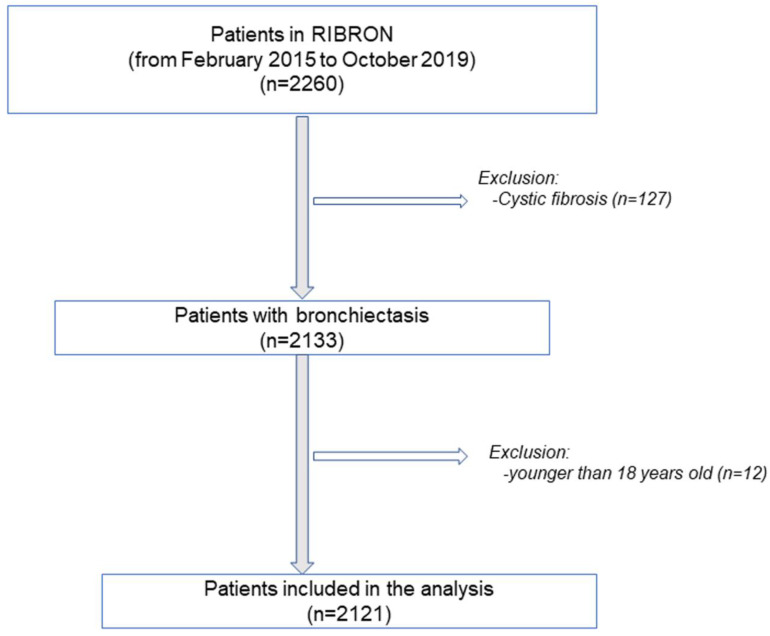
Flow-chart of the recruitment of the study patients.

The flow-chart of the patient recruitment for the purpose of the study is shown in Figure 1. Inclusion criteria were adult patients who had been diagnosed with bronchiectasis using a high-resolution computerized tomography (HRCT) [4,13,15,16,17,18]. A total number of 2121 patients were analyzed in this study. General clinical data such as anthropometry, smoking history, lung function, hemogram, inflammatory blood cells, nutritional parameters, and disease severity classification were analyzed. The patients included in the registry were all completely stable as defined by the absence of any acute exacerbation in the last four weeks or more prior to study entry. Exclusion criteria included traction bronchiectasis and/or cystic fibrosis (sweat chloride test and/or genetic confirmation), and age younger than 18 years old. The research followed the guidelines of the World Medical Association for Research in Humans (Seventh revision of the Declaration of Helsinki, Fortaleza, Brazil, 2013) [19]. Ethics approval was obtained from the Ethics Committee at the Hospital Josep Trueta Girona (# 001-2012, Hospital Universitari Dr. Josep Trueta, Girona, Spain) in all participating centers. Signed informed written consent was obtained from all the participants.

### 2.3. Study Variables and Scores

The following clinical variables and parameters were obtained from all the study patients: anthropometry (age, sex, and body mass index (BMI)), lung function, chronic colonization by *Pseudomonas aeruginosa* (PA) and by other microorganisms, radiologic extension, dyspnea, the number of acute exacerbations and hospitalizations in the previous year, the Charlson index, smoking history and nutritional status. The FACED [20], EFACED [21], and BSI [22] scores were also calculated. On the basis of the study hypothesis, the target variables were defined as follows: total number of leukocytes and neutrophils, c-reactive protein (CRP), erythrocyte sedimentation rate (ESR), hemoglobin, hematocrit, creatinine, and BMI.

### 2.4. Statistical Analysis

Quantitative variables are presented as mean (standard deviation), while qualitative variables are presented as the number of patients in each group along with the corresponding percentage. Gender differences (male versus female patients) were assessed using the T-student test for the continuous variables and the Chi-square test for the categorical variables. Disease severity scores (FACED, EFACED, and BSI) are also shown in histograms representing both male and female patients individually.

Gender differences in prespecified systemic inflammatory parameters were assessed through multiple linear regression using a robust M-estimator for the target variables to control for the presence of outliers. The clinically meaningful confounders were the following: age, smoking history (never, current o ex-smokers), COPD, acute exacerbations, hospitalizations for exacerbations in the previous year, Charlson index, colonization by PA, the number of affected lobes, forced expiratory volume in one second (FEV_1_%), and dyspnea. The multivariate regression beta coefficients are represented as black dots along with the corresponding confidence intervals in forest plots for each target variable. In the *Y*-axis all the confounder variables are plotted along with female gender, while in the *X*-axis the width of the confidence intervals is represented for all the forest plots. The zero value is represented as a dotted vertical line in each forest plot. The number of female and male patients is also provided in each forest plot.

Furthermore, with the aim to explore the potential contribution of COPD per se to the study results, the same type of analyses were performed for all the variables, in which patients with COPD were excluded (58 female and 178 male patients). Additionally, never-smoker patients were also analyzed separately with the objective to explore whether cigarette smoke may have influenced the study results.

Statistical analyses were performed using Stata 15.1 (StataCorp LLC, College Station, TX, USA). For all the analyses, the statistical significance was established as *p* < 0.05.

## 3. Results

### 3.1. Clinical Characteristics in Male and Female Patients

In the registry, a total of 1368 female (64.5%) and 753 male patients (35.5%) were included prospectively. When all the patients were considered together, males were significantly older, had a more severe disease (FACED, EFACED, and BSI scores), more comorbidities (Charlson index), a greater number of hospitalizations due to acute exacerbations, a greater level of dyspnea, and the number of packs-year was greater than in female patients (Table 1). Furthermore, male patients exhibited mild airway obstruction, better diffusion capacity, and showed a lower degree of airway trapping than female patients (Table 1). Radiological extension was similar in both groups of patients. Similar results, except for dyspnea, were observed for most of the variables when patients with concomitant COPD were excluded from the analysis (Table 2). A subanalysis of lung function status was conducted in never-smoker patients as a whole (Table 3). In this subanalysis, male patients were significantly younger than females, airflow limitation was greater among the male patients, while diffusion capacity and air trapping values were significantly worse among the female patients (Table 3).

The etiology of bronchiectasis was diverse as illustrated in Table 4. The proportions of female patients with post-infectious bronchiectasis and of unknown etiology were significantly greater than in male patients (Table 4). The proportion of COPD patients, however, was significantly greater among the latter patients than the former (Table 4).

### 3.2. Nutritional Status in Male and Female Patients

In male patients, BMI as a whole and the proportions of patients with BMI ≥ 25 kg/m^2^ were significantly higher than in female patients (Table 1). Moreover, the parameters hemoglobin, hematocrit, and creatinine levels were also significantly greater in male than in female patients (Table 1). Similar results were observed in this subanalysis after having excluded COPD patients (Table 2). No significant correlations were found between nutritional status and any of the clinical variables.

### 3.3. Inflammatory Parameters in Male and Female Patients

Compared to female patients, in male patients, total numbers of leukocytes and neutrophils were significantly higher, while those of lymphocytes and platelets were lower (Table 1). Moreover, CRP levels were also increased in male compared to female patients, while ESR levels were greater in the latter group of patients (Table 1). Similar results were observed in this subanalysis after having excluded COPD patients (Table 2). No significant correlations were found between inflammatory parameters and any of the clinical variables.

### 3.4. Degree of Disease Severity

**Figure 2:**
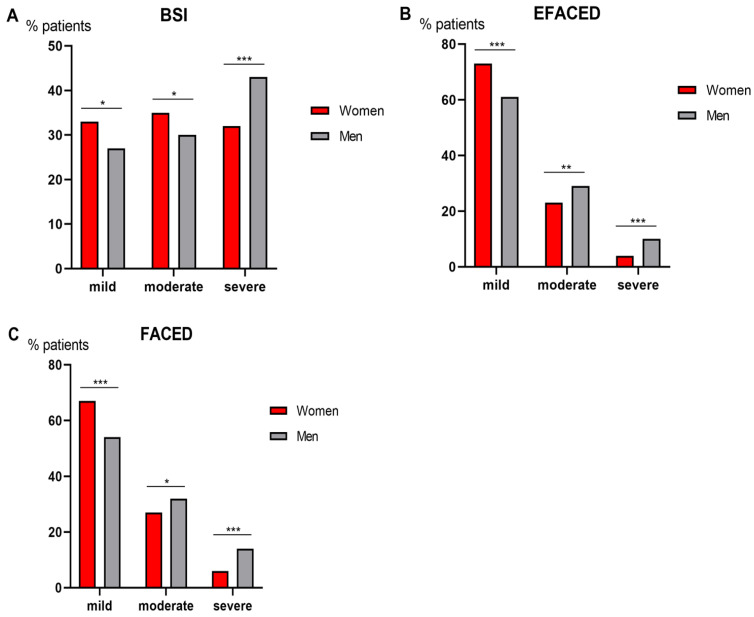
In all the patients as a whole, histograms of the proportions of patients who were classified as mild, moderate, or severe according to BSI (**A**), EFACED (**B**) and FACED (**C**) for both female (red color) and male (grey color) patients. Score subdivisions were as follows: (1) BSI: mild: 0–4, moderate: 5–8, severe: ≥9, (2) EFACED: mild: 0–3, moderate: 4–6, severe: 7–9; and (3) FACED: mild: 0–2, moderate: 3–4, severe: 5–7. Statistical significance: * *p* ≤ 0.05; ** *p* ≤ 0.01; *** *p* ≤ 0.001 between female and male patients for each score.

**Figure 3:**
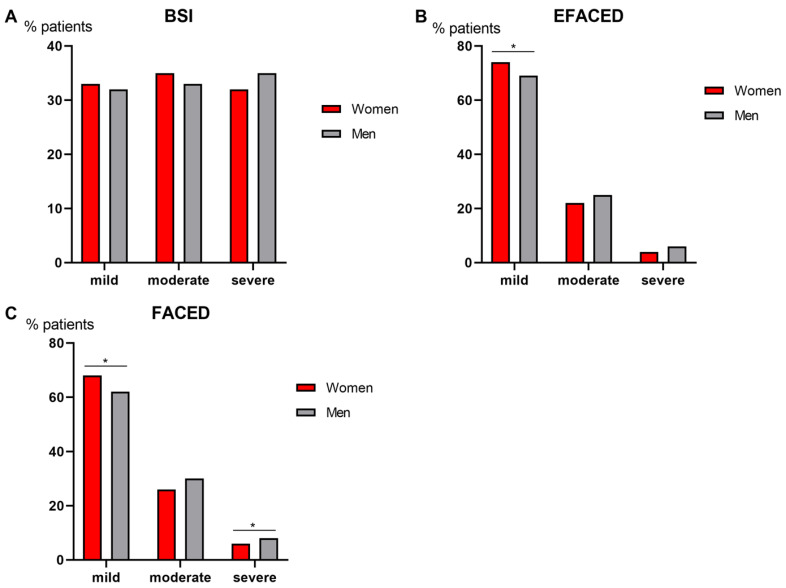
In bronchiectasis patients with no COPD, histograms of the proportions of patients who were classified as mild, moderate, or severe according to BSI (**A**), EFACED (**B**) and FACED (**C**) for both female (red color) and male (grey color) patients. Score subdivisions were as follows: (1) BSI: mild: 0–4, moderate: 5–8, severe: ≥ 9, (2) EFACED: mild: 0–3, moderate: 4–6, severe: 7–9; and (3) FACED: mild: 0–2, moderate: 3–4, severe: 5–7. Statistical significance: * *p* ≤ 0.05 between female and male patients for each score.

Compared to male patients, female patients had a less severe disease as measured by BSI score, since greater proportions of females were classified as mild and moderate (Figure 2A). When patients were classified according to EFACED and FACED, a greater proportion of female patients also had a mild disease, while a higher proportion of male patients fell into the categories moderate and severe (Figure 2B,C, respectively). In the subanalysis in which COPD patients were excluded for both female and male patients, no significant differences were seen in BSI scores (Figure 3A), while the proportions of male patients who fell into the EFACED and FACED mild category were lower than female patients (Figure 3B,C, respectively). According to FACED scores, male patients were more severe than female patients in the subanalysis (Figure 3C).

### 3.5. Multivariate Analysis of Systemic Inflammatory and Nutritional Parameters in Male and Female Patients

**Figure 4:**
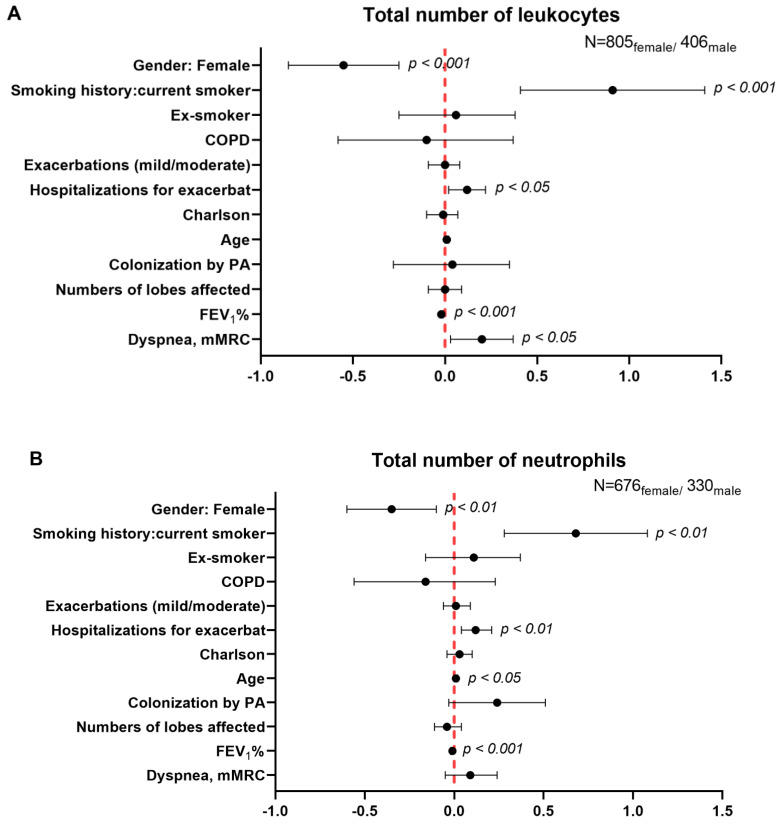

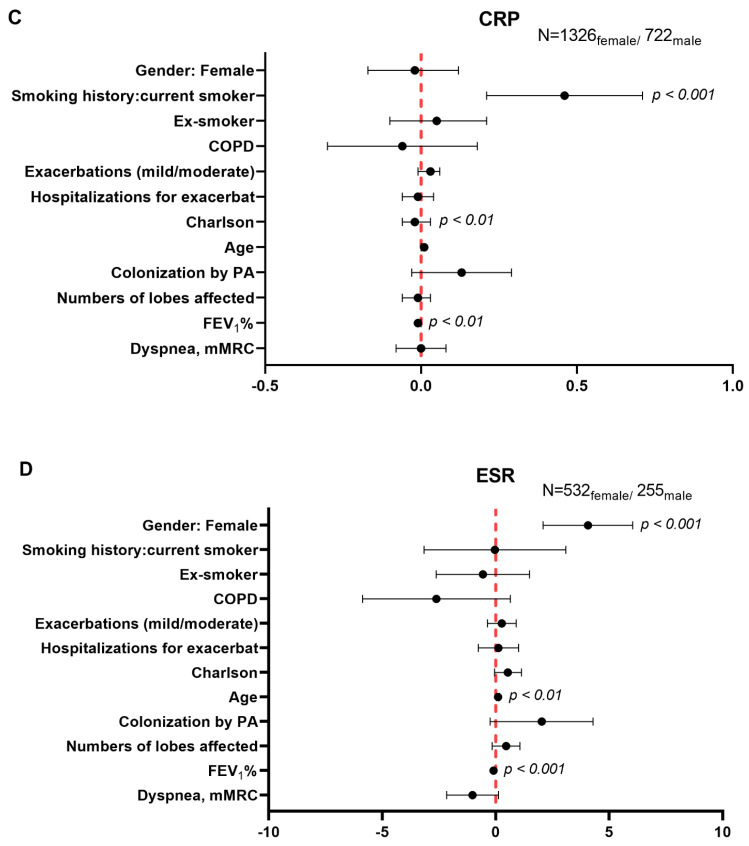
Independent associations between female gender and the following variables: total number of leukocytes (**A**), total number of neutrophils (**B**), CRP levels (**C**), ESR levels (**D**). Multivariate robust regression analysis was used to perform the analysis. Confidence intervals and statistical significance are represented in each figure panel. Beta coefficients are represented as black dots. The zero value is represented as a red dotted vertical line in each forest plot. The model was adjusted by age, smoking history (never, current, or ex-smokers), exacerbations mild-moderate, hospitalizations due to exacerbations in the previous year, Charlson Index, colonization by PA, numbers of affected lobes, FEV_1_%, and dyspnea score for each variable.

**Figure 5:**
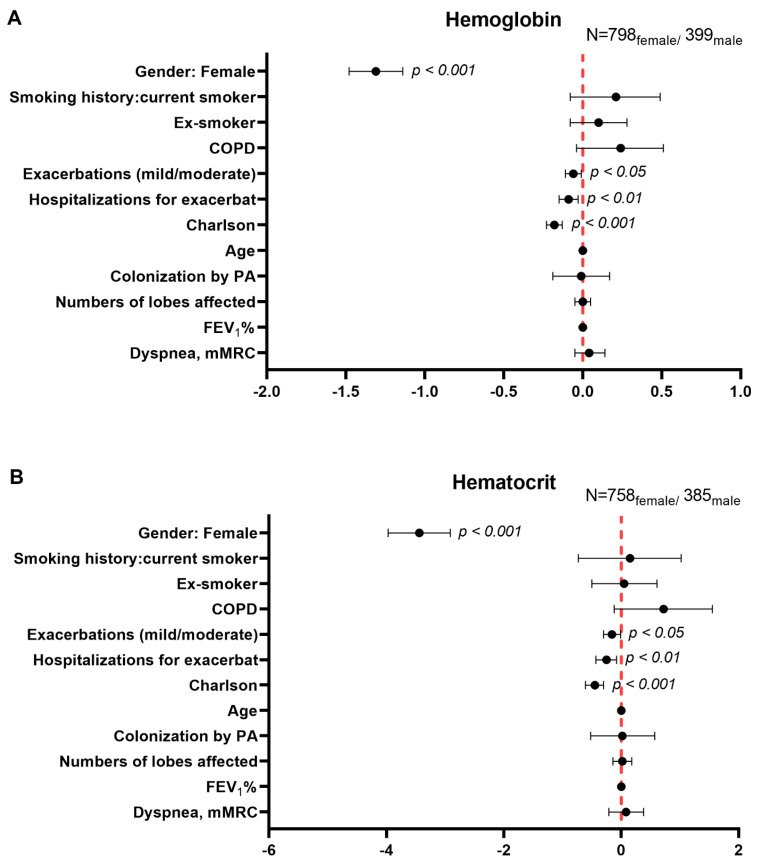

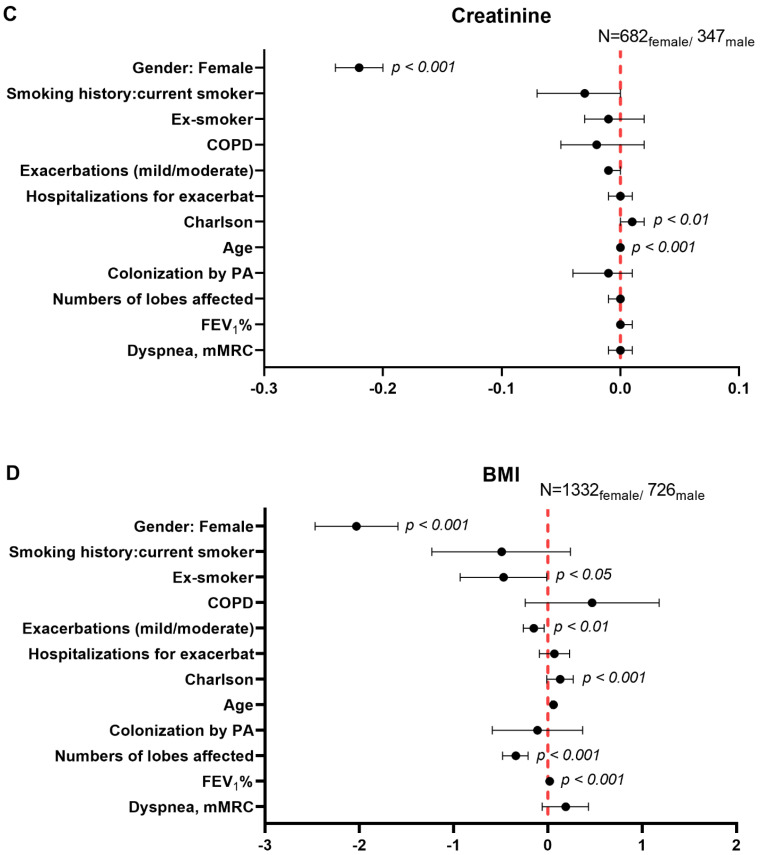
Independent associations between female gender and the following variables: hemoglobin levels (**A**), hematocrit levels (**B**), creatinine levels (**C**), BMI values (**D**). Multivariate robust regression analysis was used to perform the analysis. Confidence intervals and statistical significance are represented in each figure panel. Beta coefficients are represented as black dots. The zero value is represented as a red dotted vertical line in each forest plot. The model was adjusted by age, smoking history (never, current, or ex-smokers), exacerbations mild-moderate, hospitalizations due to exacerbations in the previous year, Charlson Index, colonization by PA, numbers of affected lobes, FEV_1_%, and dyspnea score for each variable.

Multivariate regression analysis showed that total numbers of leukocytes and neutrophils were significantly and inversely associated with female gender (β = −0.55, 95% CI: −0.85, −0.25, *p* = 0.000 and β = −0.35, 95% CI: −0.60, −0.10; *p* = 0.007, Figure 4A,B, respectively). Nonetheless, no significant associations were found between female gender and CRP levels (β = −0.02; 95% CI: −0.17, 0.12; *p* = 0.755, Figure 4C). Significant positive associations were seen between female gender and ESR levels (β = 4.07, 95% CI: 2.09, 6.04; *p* = 0.000, Figure 4D). Furthermore, negative associations were detected between hemoglobin, hematocrit, and creatinine levels, BMI, and female gender in the patients (β = −1.31, 95% CI: −1.48, −1.14; *p* = 0.000, β = −3.44, 95% CI: −3.97, −2.91; *p* = 0.000, β = −0.22; 95% CI: −0.24, −0.2; *p* = 0.000, and β = −2.03; 95% CI: −2.47, −1.59; *p* = 0.000), Figure 5A–D, respectively). Notably, all these differences were independent of the presence of COPD (Figure 4A–D and Figure 5A–D).

## 4. Discussion

In the present study, the proportions of female patients included in the registry were twice as great as those of male patients. Interestingly, female patients had a less severe disease as measured by all three indices of disease severity, a lower degree of airway obstruction, worse diffusion capacity and airway trapping, even when non-COPD or never-smoker patients were analyzed separately, a greater prevalence of idiopathic etiology, better nutritional markers, and lower levels of inflammatory parameters except for ESR. Multivariate regression analysis evidenced that strong relationships were found between female gender and the variables total numbers of leukocytes and neutrophils, ESR, hemoglobin, hematocrit, creatinine, and BMI. In view of these findings, the study hypothesis has been confirmed to a great extent.

In the current investigation, male patients exhibited greater scores of disease severity along with a higher degree of airway obstruction, while showing better lung diffusion capacity and lower air trapping than female patients. These findings were confirmed in the subanalysis after having excluded the COPD patients in both groups. These results are somehow in line with those previously reported [13], in which airflow limitation was less prominent in female patients with bronchiectasis than in men. In keeping with, disease severity scores were increased in male patients compared to females, despite that the degree of radiological extension was similar between both genders [13]. However, airway trapping and diffusion capacity were significantly better in male patients than in females in all the analyses: total population, when COPD patients were excluded, and when only never-smoker patients were analyzed separately. In fact, it has been demonstrated that respiratory function differs between males and females probably due to anatomical and size differences [23]. These results warrant further attention in future investigations.

In the present study, substantial differences were observed between female and male patients as to the nutritional parameters assessed in the registry. As such, BMI was significantly greater in the male patients than in the females. Furthermore, when patients were subdivided according to three different degrees of BMI, a greater percentage of male patients fell into the category of a BMI > 25 kg/m^2^ than female patients. These are relevant findings that suggest that BMI may influence disease morbidity and prognosis as reported in another large cohort of patients with bronchiectasis [24]. In the multivariate regression analysis, BMI was also significantly associated with female gender. Hence, these results confirm the relevance of BMI as a discriminatory end-point between male and female patients with bronchiectasis. Lastly, it should also be mentioned that greater numbers and proportions of female patients (173, 13%) fell into the category of BMI < 20 kg/m^2^ than in male patients (45, 6%). These results were also confirmed in the subanalysis, in which the COPD patients were excluded.

Importantly, levels of the nutritional parameters—hemoglobin, hematocrit, and creatinine—were significantly greater in the male than in the female patients. Multivariate regression analyses also confirmed the associations seen between each of those blood parameters with female gender. Differences in hemoglobin levels were also reported in male and female patients with COPD, in whom this blood parameter was demonstrated to have predictive value during hospitalizations and at hospital discharge [25,26]. As far as we are concerned, these are novel findings in patients with bronchiectasis. Creatinine is a marker of renal function, since it is excreted by the kidneys and is a product of muscle protein metabolism, thus it can be also used as an indicator of nutritional status in patients. In the current investigation, levels of creatinine were significantly greater in male patients than in females, despite that in both groups, levels of this marker were within the normal ranges for men and women, respectively. The subanalysis without the COPD patients also revealed similar results, suggesting that the increase in plasma creatinine levels was independent of this disease in this series of bronchiectasis patients. In the multivariate regression analyses, the significant differences in creatinine levels between male and female patients were confirmed. These findings suggest that muscle protein metabolism was higher among the male patients. Similar findings were also reported in a cohort of patients with COPD [27].

Total levels of leukocytes and neutrophils were significantly increased in male patients compared to females in both types of analyses, when patients were considered as a whole and after excluding those with concomitant COPD. Importantly, white blood cell counts were shown to significantly correlate with current smoking status and COPD severity as well as to predict poor lung function and quality of life in the patients [28]. Indeed, it was proposed that white blood cell counts may be used as a reliable biomarker of disease progression and severity among patients with COPD [28]. In the present study, the multivariate regression analyses evidenced that both total numbers of leukocytes and neutrophils were statistically different between male and female patients. The presence of COPD did not affect the encountered differences between male and female patients regarding the numbers of white blood cells.

The number of blood lymphocytes may be another potential biomarker of nutritional status in chronic respiratory patients [29,30]. In a previous investigation [29], no significant differences in lymphocyte counts were detected between malnourished and well-nourished COPD patients. In the current study, the percentage of lymphocytes was lower in male than in female bronchiectasis patients. Nonetheless, as the values were within the normal ranges (20–40%) in both groups, we believe that the contribution of sex-related differences in lymphocyte counts to alterations in the nutritional status of the study patients was probably negligible.

Furthermore, significant differences were also detected in other blood parameters such as CRP and ESR between female and male patients. Nonetheless, those differences were not confirmed in the multivariate analyses for CRP, thus implying that factors other than gender-related issues may account for the differences seen in CRP levels between the two groups of patients. Interestingly, ESR levels were within normal ranges in both male and female patients and as previously demonstrated in a population-based study [31], values were also greater in females than in males in the present investigation.

## 5. Study Critique

The current investigation is based on a large number of well-characterized patients that were recruited from different participating centers in the registry. Having said that, a few limitations need to be reported. This is a clinical descriptive study in which differences between male and female patients with bronchiectasis have been demonstrated in several nutritional and systemic inflammation biomarkers. Gender-related differences as to the potential predictive value of the target biomarkers, however, have not been explored in the current investigation. Follow-up studies will be required in order to show the relative weight of each biomarker in disease progression and severity in female and male patients with bronchiectasis. Despite these concerns, the present study will serve as the basis for the design of follow-up investigations intended to address such a relevant question in the near future. Finally, an external validation of our results would be also desirable in future investigations.

## 6. Conclusions

Our study shows that several nutritional parameters and inflammatory biomarkers may be reliable indicators of gender-related differences in patients with bronchiectasis. The reported findings herein deserve further attention in follow-up investigations in which the potential predictive value of those biomarkers should be thoroughly explored.

## Figures and Tables

**Table 1:** Clinical parameters and nutritional and inflammatory status of all the study patients: females and males.

	Women	Men
	N = 1368	N = 753
**Anthropometric variables, ** **$\bar{x}\;$** **(SD)**		
Age (years)	68.5 (14.4)	69.9 (15.3) *
**Disease severity, ** **$\bar{x}\;$** **(SD)**		
FACED score	1.9 (1.6)	2.4 (1.8) ***
EFACED score	2.4 (2)	3.1 (2.3) ***
BSI score	7.2 (4.3)	8.1 (4.8) ***
# exacerbations, previous year	1.6 (1.9)	1.5 (1.8)
Hospitalizations for exacerbations in the previous year	0.5 (1.2)	0.7 (1.5) ***
Charlson Index	1.6 (1.2)	2.2 (1.8) ***
Radiological extension	2.8 (1.4)	2.8 (1.5)
Dyspnea, mMRC	1.9 (0.9)	2.1 (1.0) ***
Chronic colonization by PA, N (%)	311 (22.7)	189 (25.1)
**Smoking history**		
Current smokers, N (%)	109 (8)	69 (9)
Ex-smokers, N (%)	296 (22)	409 (54) ***
Never smokers, N (%)	963 (70)	275 (37) ***
Packs-year, $\bar{x}$ (SD)	24 (21)	39 (31) ***
**Lung function testing, ** **$\bar{x}\;$** **(SD)**		
FEV_1_, % predicted	77 (24)	68 (24) ***
FVC, % predicted	86 (23)	79 (19) ***
FEV_1_/FVC, %	71 (12)	65 (15) ***
DL_CO_, % predicted	82 (23)	86 (25)
K_CO_, % predicted	78 (35)	87 (37) **
RV, % predicted	140 (42)	135 (56)
TLC, % predicted	104 (19)	100 (21) *
RV/TLC, %	53 (13)	47 (12) ***
**Nutritional status**		
$\text{BMI},\;\text{kg}/m^{2},\;\bar{x}$ (SD)	25 (5)	27 (4) ***
BMI grade kg/m^2^		
<20, N (%)	173 (13)	45 (6) ***
20–25, N (%)	580 (42)	231 (31) ***
≥25, N (%)	614 (45)	476 (63) ***
$\text{Hemoglobin},\;g/\mathrm{dL},\;\bar{x}$ (SD)	13.21 (1.29)	14.31 (1.75) ***
$\text{Hematocrit},\;\%,\;\bar{x}$ (SD)	40.38 (3.8)	43.4 (4.94) ***
$\text{Creatinine},\;\text{mg}/\text{dL},\;\bar{x}$ (SD)	0.75 (0.29)	1.01 (0.55) ***
$\text{Total}\;\text{proteins},\;g/\text{dL},\;\bar{x}$ (SD)	7.04 (0.61)	6.99 (0.65)
$\text{Albumin},\;g/\text{dL},\;\bar{x}$ (SD)	4.19 (0.42)	4.18 (0.49)
**Systemic inflammatory markers**		
$\text{Systemic}\;\text{inflammatory}\;\text{cells},\;\bar{x}$ (SD)		
Total number of leukocytes, cells/uL	7.40 (3.67) × 10^3^	8.43 (4.01) × 10^3^ ***
Total number of neutrophils, cells/uL	4.46 (2.41) × 10^3^	5.26 (2.75) × 10^3^ ***
Neutrophils, %	58.96 (12.42)	62.47 (12.36) ***
Total number of lymphocytes, cells/uL	2.12 (2.43) × 10^3^	2.08 (2.88) × 10^3^
Lymphocytes, %	29.29 (11.18)	25.59 (10.72) ***
Total number of eosinophils, cells/uL	0.20 (0.20) × 10^3^	0.21 (0.22) × 10^3^
Eosinophils, %	2.85 (2.73)	2.82 (2.66)
Platelets, cells/uL	260 (77) × 10^3^	241 (75) × 10^3^ ***
$\mathrm{Acute}-\mathrm{phase}\;\mathrm{reactants},\;\bar{x}$ (SD)		
Alpha-1 antitrypsin, mg/dL	135.85 (42.41)	130.87 (39.79)
CRP, mg/dL	5.30 (12.47)	6.01 (13.79) *
Fibrinogen, mg/dL	434.12 (136.95)	433.64 (147.24)
ESR, mm/h	21.00 (18.61)	15.29 (14.79) ***

Continuous variables are presented as mean (standard deviation), while categorical variables are presented as the number of patients in each group along with the percentage for the study group. Definition of abbreviations: N, number; #, number; kg, kilograms; m, meters; BSI: bronchiectasis severity index, BMI, body mass index; FEV_1_, forced expiratory volume in the first second; FVC, forced vital capacity; RV, residual volume; TLC, total lung capacity; DLco, carbon monoxide transfer; K_CO_, Krogh transfer factor; FACED: F, FEV_1;_ A, Age; C, Chronic colonization by *Pseudomonas aeruginosa*; E, radiological extension; D, dyspnea; mMRC, modified Medical Research Council; PA: *Pseudomonas aeruginosa*; CRP, C-reactive protein; ESR, erythrocyte sedimentation rate; kg, kilogram; g, grams; dL, deciliter; uL, microliter; mg, milligrams; mm, millimeters; h, hour. Statistical analyses and significance: * *p* < 0.05; ** *p* < 0.01; *** *p* < 0.001 between men and women patients.

**Table 2:** Clinical parameters and nutritional and inflammatory status of bronchiectasis patients excluding those with COPD: females and males.

	Women	Men
	N = 1310	N = 575
**Anthropometric variables, ** **$\bar{x}\;$** **(SD)**		
Age (years)	68.3 (14.5)	67.4 (16)
**Disease severity, ** **$\bar{x}\;$** **(SD)**		
FACED score	1.9 (1.6)	2.1 (1.7) **
EFACED score	2.4 (2)	2.7 (2.1) **
BSI score	7.1 (4.3)	7.2 (4.4)
# exacerbations, previous year	1.6 (1.9)	1.5 (1.8)
Hospitalizations for exacerbations in the previous year	0.5 (1.2)	0.5 (1.1)
Charlson Index	1.6 (1.2)	2 (1.7) ***
Radiological extension	2.8 (1.4)	2.8 (1.5)
Dyspnea, mMRC	1.8 (0.9)	1.9 (0.9)
Chronic colonization by PA, N (%)	302 (23.1)	139 (24.2)
**Smoking history**		
Current smokers, N (%)	87 (7)	41 (7)
Ex-smokers, N (%)	267 (20)	264 (46) ***
Never smokers, N (%)	956 (73)	270 (47) ***
Packs-year, $\bar{x}$ (SD)	21 (19)	29 (24) ***
**Lung function testing, ** **$\bar{x}\;$** **(SD)**		
FEV_1_, % predicted	78 (24)	72 (24) ***
FVC, % predicted	87 (23)	81 (19) ***
FEV_1_/FVC, %	72 (12)	69 (14) ***
DL_CO_, % predicted	84 (22)	89 (25) *
K_CO_, % predicted	78 (35)	89 (38) **
RV, % predicted	138 (40)	128 (54)
TLC, % predicted	103 (19)	97 (19) **
RV/TLC, %	53 (13)	45 (13) ***
**Nutritional status**		
BMI, kg/m^2^, $\bar{x}$ (SD)	25 (5)	27 (4) ***
BMI grade kg/m^2^		
<20, N (%)	165 (13)	35 (6) ***
20–25, N (%)	556 (42)	178 (31) ***
≥25, N (%)	588 (45)	362 (63) ***
Hemoglobin, g/dL, $\bar{x}$ (SD)	13.2 (1.28)	14.34 (1.69) ***
Hematocrit, %, $\bar{x}$ (SD)	40.34 (3.75)	43.45 (4.71) ***
Creatinine, mg/dL, $\bar{x}$ (SD)	0.75 (0.3)	1.03 (0.62) ***
Total proteins, g/dL, $\bar{x}$ (SD)	7.05 (0.6)	7.06 (0.63)
Albumin, g/dL, $\bar{x}$ (SD)	4.21 (0.41)	4.25 (0.43)
**Systemic inflammatory markers**		
Systemic inflammatory cells, $\bar{x}$ (SD)		
Total number of leukocytes, cells/uL	7.38 (3.72) × 10^3^	8.05 (3) × 10^3^ **
Total number of neutrophils, cells/uL	4.43 (2.39) × 10^3^	5.03 (2.56) × 10^3^ **
Neutrophils, %	58.8 (12.33)	61.77 (11.76) ***
Total number of lymphocytes, cells/uL	2.13 (2.48) × 10^3^	1.93 (0.79) × 10^3^
Lymphocytes, %	29.42 (11.15)	26.4 (10.05) ***
Total number of eosinophils, cells/uL	0.19 (0.2) × 10^3^	0.21 (0.22) × 10^3^
Eosinophils, %	2.84 (2.61)	2.86 (2.64)
Platelets, cells/uL	260 (77) × 10^3^	242 (76) × 10^3^ **
Acute-phase reactants, $\bar{x}$ (SD)		
Alpha-1 antitrypsin, mg/dL	135.96 (43.24)	127.6 (40.1) *
CRP, mg/dL	5.38 (12.63)	5.38 (12.3)
Fibrinogen, mg/dL	432.01 (136.49)	420.73 (144.98)
ESR, mm/h	21.15 (18.65)	14.69 (13.56) ***

Continuous variables are presented as mean (standard deviation), while categorical variables are presented as the number of patients in each group along with the percentage for the study group. Definition of abbreviations: N, number; #, number; kg, kilograms; m, meters; BSI: bronchiectasis severity index, BMI, body mass index; FEV_1_, forced expiratory volume in the first second; FVC, forced vital capacity; RV, residual volume; TLC, total lung capacity; DLco, carbon monoxide transfer; K_CO_, Krogh transfer factor; FACED: F, FEV_1;_ A, Age; C, Chronic colonization by *Pseudomonas aeruginosa*; E, radiological extension; D, dyspnea; mMRC, modified Medical Research Council scale; PA, *Pseudomonas aeruginosa*; CRP, C-reactive protein; ERS, erythrocyte sedimentation rate; kg, kilogram; g, grams; dL, deciliter; uL, microliter; mg, milligrams; mm, millimeters; h, hour. Statistical analyses and significance: * *p* < 0.05; ** *p* < 0.01; *** *p* < 0.001 between men and women patients.

**Table 3:** Lung function in never-smoker patients: females and males.

	Women	Men
	N = 963	N = 275
**Anthropometric variables, ** **$\bar{x}\;$** **(SD)**		
Age (years)	69.7 (15.4)	64.7 (18) ***
**Lung function testing, ** **$\bar{x}\;$** **(SD)**		
FEV_1_, % predicted	77 (25)	73 (23) *
FVC, % predicted	86 (24)	81 (18) ***
FEV_1_/FVC, %	71 (12)	69 (14) *
DL_CO_, % predicted	85 (23)	90 (23)
K_CO_, % predicted	80 (35)	94 (38) *
RV, % predicted	135 (41)	125 (54)
TLC, % predicted	101 (19)	97 (20)
RV/TLC, %	53 (14)	44 (14) ***

Continuous variables are presented as mean (standard deviation) for the study group. Definition of abbreviations: FEV1, forced expiratory volume in the first second; FVC, forced vital capacity; RV, residual volume; TLC, total lung capacity; DLco, carbon monoxide transfer; KCO, Krogh transfer factor. Statistical analyses and significance: * *p* < 0.05; *** *p* < 0.001 between male and female patients.

**Table 4:** Etiology of non-CF bronchiectasis patients.

Etiology, N, %	Total	Women	Men
**Post-infectious**	865, 40.8%	603, 44.1%	262, 34.8% ***
Tuberculosis	262, 12.4%	167, 12.2%	95, 12.6%
Childhood infections	160, 7.5%	127, 9.3%	33, 4.4 ***
Necrotizing pneumonia	53, 2.5%	38, 2.8%	15, 2.0%
Non-tuberculous mycobacteria	17, 0.8%	13, 1.0%	4, 0.5%
Fungal infections	8, 0.4%	4, 0.3%	4, 0.5%
Others	1621, 76.4%	1019, 74.5%	602, 79.9% **
**Unknown etiology**	382, 18%	285, 20.8%	97, 12.9% ***
**COPD**	236, 11.1%	58, 4.2%	178, 23.6% ***
**Asthma**	179, 8.4%	128, 9.4%	51, 6.8% *
**Systemic disorders**	164, 7.7%	102, 7.5%	62, 8.2%
**Immunodeficiencies**	85, 4.0%	53, 3.9%	32, 4.2%
**Inflammatory pneumonitis**	37, 1.7%	29, 2.1%	8, 1.1%
**Inflammatory bowel diseases**	17, 0.8%	11, 0.8%	6, 0.8%
**Congenital malformations**	15, 0.7%	6, 0.4%	9, 1.2%
**Obliterative bronchiolitis**	11, 0.5%	8, 0.6%	3, 0.4%
**Hyperimmune response**	6, 0.3%	4, 0.3%	2, 0.3%
**Vasculitis**	4, 0.2%	1, 0.1%	3, 0.4%
**Other etiologies**	120, 5.7%	80, 5.8%	40, 5.3%

Absolute number of patients and percentage for each etiologic condition. Definition of abbreviations: N, number; %, percentage; COPD: chronic obstructive pulmonary disease. Statistical analyses and significance: * *p* < 0.05; ** *p* < 0.01; *** *p* < 0.001 between the two groups of patients.

## Data Availability

The datasets are available from the corresponding authors upon reasonable request.

## References

[1] Martinez-Garcia M.A., Agustí A. (2019). Heterogeneidad y complejidad del síndrome bronquiectasias: Un reto pendiente. Arch. Bronconeumol..

[2] Martinez-Garcia M.A., de la Rosa D., Cantón R., Olveira C., Máiz-Carro L., Girón R., Prados C., Blanco M. (2019). Bronquiectasias: Cuando la evidencia científica publicada no resulta suficiente. Arch. Bronconeumol..

[3] Martínez-García M.A., Olveira C., Máiz L., Girón R.M., Prados C., de la Rosa D., Blanco M., Agustí A. (2019). Bronchiectasis: A Complex, Heterogeneous Disease. Arch. Bronconeumol..

[4] Martínez-García M.Á., Máiz L., Olveira C., Girón R.M., de la Rosa D., Blanco M., Cantón R., Vendrell M., Polverino E., de Gracia J. (2018). Spanish Guidelines on the Evaluation and Diagnosis of Bronchiectasis in Adults. Arch. Bronconeumol..

[5] Morrissey B.M., Harper R.W. (2004). Bronchiectasis: Sex and gender considerations. Clin. Chest Med..

[6] Vidaillac C., Yong V.F.L., Jaggi T.K., Soh M.M.-M., Chotirmall S.H. (2018). Gender differences in bronchiectasis: A real issue?. Breathe.

[7] Sánchez-Muñoz G., Lopez-De-Andrés A., Hernández-Barrera V., Jiménez-García R., Pedraza-Serrano F., Puente-Maestu L., De Miguel-Díez J. (2019). Bronchiectasis in patients hospitalized with acute exacerbation of COPD in Spain: Influence on mortality, hospital stay, and hospital costs (2006–2014) according to gender. PLoS ONE.

[8] Pinkerton K.E., Harbaugh M., Han M.L.K., Le Saux C.J., Van Winkle L.S., Martin W.J., Kosgei R.J., Carter E.J., Sitkin N., Smiley-Jewell S.M. (2015). Women and lung disease: Sex differences and global health disparities. Am. J. Respir. Crit. Care Med..

[9] Nick J.A., Chacon C.S., Brayshaw S.J., Jones M.C., Barboa C.M., St. Clair C.G., Young R.L., Nichols D.P., Janssen J.S., Huitt G.A. (2010). Effects of gender and age at diagnosis on disease progression in long-term survivors of cystic fibrosis. Am. J. Respir. Crit. Care Med..

[10] Demko C.A., Byard P.J., Davis P.B. (1995). Gender differences in cystic fibrosis: Pseudomonas aeruginosa infection. J. Clin. Epidemiol..

[11] Menéndez R., Méndez R., Polverino E., Rosales-Mayor E., Amara-Elori I., Reyes S., Posadas T., Fernández-Barat L., Torres A. (2017). Factors associated with hospitalization in bronchiectasis exacerbations: A one-year follow-up study. Respir. Res..

[12] He M., Zhu M., Wang C., Wu Z., Xiong X., Wu H., Cheng D., Ji Y. (2020). Prognostic performance of the FACED score and bronchiectasis severity index in bronchiectasis: A systematic review and meta-analysis. Biosci. Rep..

[13] Martinez-García M.A., Villa C., Dobarganes Y., Girón R., Maíz L., García-Clemente M., Sibila O., Golpe R., Rodríguez J., Barreiro E. (2021). RIBRON: The spanish Online Bronchiectasis Registry. Characterization of the First 1912 Patients. Arch. Bronconeumol..

[14] Von Elm E., Altman D.G., Egger M., Pocock S.J., Gøtzsche P.C., Vandenbroucke J.P. (2008). The Strengthening the Reporting of Observational Studies in Epidemiology (STROBE) statement: Guidelines for reporting observational studies. J. Clin. Epidemiol..

[15] Chalmers J.D., Chang A.B., Chotirmall S.H., Dhar R., McShane P.J. (2018). Bronchiectasis. Nat. Rev. Dis. Prim..

[16] Posadas T., Oscullo G., Zaldivar E., Villa C., Dobarganes Y., Girón R., Olveira C., Maíz L., García-Clemente M., Sibila O. (2021). C-Reactive Protein Concentration in Steady-State Bronchiectasis: Prognostic Value of Future Severe Exacerbations. Data From the Spanish Registry of Bronchiectasis (RIBRON). Arch. Bronconeumol..

[17] Polverino E., Goeminne P.C., McDonnell M.J., Aliberti S., Marshall S.E., Loebinger M.R., Murris M., Cantón R., Torres A., Dimakou K. (2017). European Respiratory Society guidelines for the management of adult bronchiectasis. Eur. Respir. J..

[18] Aliberti S., Masefield S., Polverino E., De Soyza A., Loebinger M.R., Menendez R., Ringshausen F.C., Vendrell M., Powell P., Chalmers J.D. (2016). Research priorities in bronchiectasis: A consensus statement from the EMBARC Clinical Research Collaboration. Eur. Respir. J..

[19] Shrestha B., Dunn L. (2020). The Declaration of Helsinki on Medical Research involving Human Subjects: A Review of Seventh Revision. J. Nepal Health Res. Counc..

[20] Martinez-Garcia M.A., de Gracia J., Vendrell Relat M., Giron R.-M., Maiz Carro L., de la Rosa Carrillo D., Olveira C. (2014). Multidimensional approach to non-cystic fibrosis bronchiectasis: The FACED score. Eur. Respir. J..

[21] Martinez-Garcia M.A., Athanazio R.A., Girón R.M., Máiz-Carro L., de la Rosa D., Olveira C., de Gracia J., Vendrell M., Prados-Sánchez C., Gramblicka G. (2017). Predicting high risk of exacerbations in bronchiectasis: The E-FACED score. Int. J. Chron. Obstruct. Pulmon. Dis..

[22] Chalmers J.D., Goeminne P., Aliberti S., McDonnell M.J., Lonni S., Davidson J., Poppelwell L., Salih W., Pesci A., Dupont L.J. (2014). The bronchiectasis severity index an international derivation and validation study. Am. J. Respir. Crit. Care Med..

[23] Lomauro A., Aliverti A. (2018). Sex differences in respiratory function. Breathe.

[24] Qi Q., Li T., Li J.C., Li Y. (2015). Association of body mass index with disease severity and prognosis in patients with non-cystic fibrosis bronchiectasis. Braz. J. Med. Biol. Res..

[25] Toft-Petersen A.P., Torp-Pedersen C., Weinreich U.M., Rasmussen B.S. (2016). Association between hemoglobin and prognosis in patients admitted to hospital for COPD. Int. J. COPD.

[26] Xu L., Chen Y., Xie Z., He Q., Chen S., Wang W., Liu G., Liao Y., Lu C., Hao L. (2019). High hemoglobin is associated with increased in-hospital death in patients with chronic obstructive pulmonary disease and chronic kidney disease: A retrospective multicenter population-based study. BMC Pulm. Med..

[27] Durmus Kocak N., Sasak G., Akturk U.A., Akgun M., Boga S., Sengul A., Gungor S., Arinc S. (2016). Serum uric acid levels and uric acid/creatinine ratios in stable chronic obstructive pulmonary disease (COPD) patients: Are these parameters efficient predictors of patients at risk for exacerbation and/or severity of disease?. Med. Sci. Monit..

[28] Koo H.-K., Kang H.K., Song P., Park H.K., Lee S.-S., Jung H. (2017). Systemic White Blood Cell Count as a Biomarker Associated with Severity of Chronic Obstructive Lung Disease. Tuberc. Respir. Dis..

[29] Thorsdottir I., Gunnarsdottir I., Eriksen B. (2001). Screening Method Evaluated by Nutritional Status Measurements can be Used to Detect Malnourishment in Chronic Obstructive Pulmonary Disease. J. Am. Diet. Assoc..

[30] Keller U. (2019). Nutritional Laboratory Markers in Malnutrition. J. Clin. Med..

[31] Alende-Castro V., Alonso-Sampedro M., Vazquez-Temprano N., Tuñez C., Rey D., García-Iglesias C., Sopeña B., Gude F., Gonzalez-Quintela A. (2019). Factors influencing erythrocyte sedimentation rate in adults. Medicine.

